# A Case of Sustained Ventricular Tachycardia Secondary to a Cardiac Fibroma in a Patient With Gorlin Syndrome

**DOI:** 10.7759/cureus.82538

**Published:** 2025-04-18

**Authors:** Danhue Moodie, Ujjawal Paudel, Joud Fahed, Wongelawit Zerihun, Kaushal Sigdel

**Affiliations:** 1 Department of Internal medicine, Ascension Saint Agnes Hospital, Baltimore, USA; 2 Department of Internal Medicine, Ascension Saint Agnes Hospital, Baltimore, USA

**Keywords:** cardiac fibroma, computed tomography coronary angiogram, gorlin-goltz syndrome, sustanied ventricular tachycardia, transthoracic echocardiogram

## Abstract

Cardiac fibromas are a rare but important cause of ventricular tachycardia. They are known to be more arrhythmogenic than other cardiac tumors, which require definitive management with excision of the mass to reduce the risk of recurrent arrhythmias and sudden cardiac death.

We present a case of a 21-year-old male with Gorlin syndrome and cardiac fibroma presenting with chest discomfort and shortness of breath. His electrocardiogram (ECG) showed stable sustained ventricular tachycardia, initially resistant to intravenous (IV) amiodarone; however, the patient subsequently converted to normal sinus rhythm after the administration of IV metoprolol. The patient underwent imaging investigations, including an echocardiogram and computed tomography (CT) coronary angiogram. These imaging modalities showed a solid mass measuring 7.2 x6.3 x 5.8 cm in the superior aspect of the heart. It involved the anterior wall of the left and right ventricles along the mid and basal segments with extension into the interventricular septum. The coronary arteries had no luminal irregularities and normal anatomical positions. After consultation with his cardiologist, the patient was offered the placement of an automated implantable cardioverter defibrillator; however, he declined to have it placed. He ultimately agreed to pharmacological management, and he was discharged on low-dose metoprolol.

## Introduction

Ventricular tachycardias have many causes, ranging from electrolyte abnormalities and genetic channelopathies to structural abnormalities. In this case, our patient has a history of Gorlin syndrome, which is a genetic condition that predisposes patients to tumor formation. It is a condition caused by a mutation of the *PTCH* tumor suppressor gene, which leads to the formation of a variety of neoplasms, including cardiac fibromas, such as in our patient. Cardiac fibromas are usually common in children and are extremely rare primary heart tumors in adults, representing only around 1% of all primary cardiac tumors in the adult age group [1]. Compared to other more common cardiac tumors, fibromas are the most arrhythmogenic, with around 30% causing ventricular tachycardias [1,2]. Due to this characteristic, patients are at an increased risk of sudden cardiac death, and surgical resection or placement of an automated implantable cardioverter defibrillator (AICD) is paramount in reducing this risk. Notably, they tend to be asymptomatic, as in our patient, who also did not have any symptoms of exercise intolerance. Herein, we describe the case of a young adult presenting with ventricular arrhythmia secondary to a cardiac fibroma.

## Case presentation

A 21-year-old male with a history of Gorlin syndrome with congenital left ventricular mass presented with chest discomfort, palpitations, nausea, and one episode of vomiting starting on the day of presentation. He reported that these symptoms were ongoing for 3 hours before arrival to the Emergency Department (ED). Initial vitals in the ED showed a temperature of 36°Celsius, a pulse rate of 200 beats per minute, respiratory rate of 20 breaths per minute, and blood pressure of 91/67 mmHg. He was saturating at 100% on room air. The cardiac monitor showed sustained ventricular tachycardia.

On examination, he was in mild cardiopulmonary distress. His lungs were clear to auscultation. His cardiovascular examination was significant for marked tachycardia; however, no murmurs were heard. His abdomen was soft, flat, and non-tender. He was neurologically intact.

**Figure 1:**
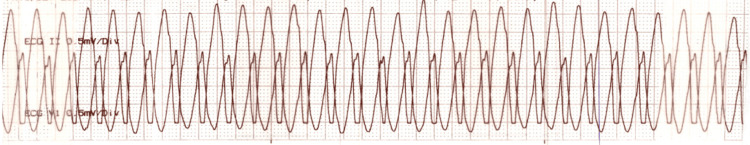
Cardiac monitor strip showing wide complex tachycardia, likely to be ventricular tachycardia, with a rate of approximately 210 beats per minute

**Figure 2:**
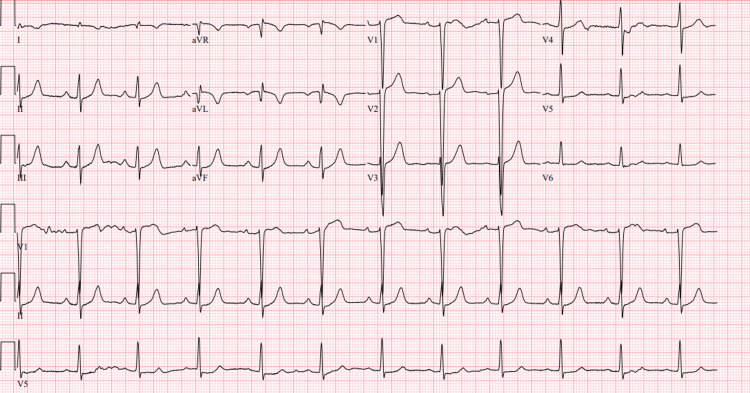
ECG showing sinus rhythm after IV amiodarone and metoprolol boluses

On admission, the cardiac monitor showed sustained ventricular tachycardia around 210 beats per minute (bpm) (Figure 1). He was given amiodarone 150 mg IV bolus followed by infusion. He required another bolus of IV amiodarone 150 mg; however, his heart rate remained in the range of 200 to 210 bpm. In an effort to achieve rate control, 5 mg of IV metoprolol was given. The patient eventually converted to sinus rhythm (Figure 2). Initial laboratory workup included complete blood count, basic metabolic panel, and troponins. The labs were significant for leukocytosis of 14.4x 10^9^/L, creatinine level of 1.3 mg/dL, and two troponin values of 0.05 ng/mL 2 hours apart. The elevated leukocytosis was thought to be reactive as there was no other evidence of infection. Additionally, his magnesium level was 2 mg/dL and brain natriuretic peptide level was 20 pg/mL (Table 1).

**Table 1:** Lab test results with reference ranges.

Test	Result	Reference range
White blood cell count	14.4x 10^9^ cells/L	4–11.0 x 10^9^ cells/L
Creatinine	1.3 mg/dL	0.72–1.25 mg/L
Troponin	0.05 ng/mL	0–0.03 ng/mL
Magnesium	2.0 mg/mL	1.6–2.6 ng/mL
Brain natriuretic peptide	20 pg/mL	0–100 pg/mL

**Figure 3:**
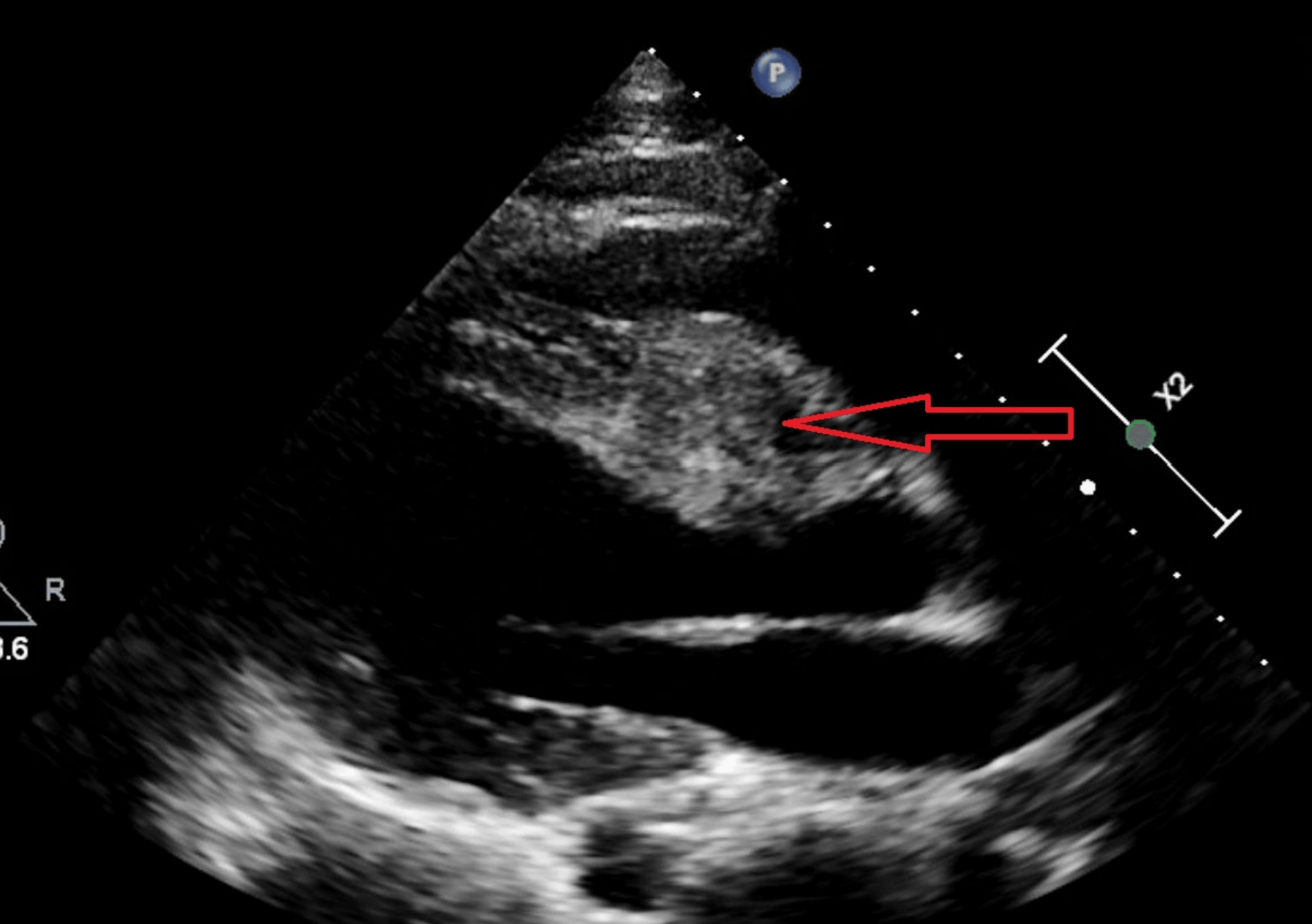
Transthoracic echocardiogram (parasternal long axis view) showing a 5-cm interventricular septal mass

Transthoracic echocardiogram (TTE) showed a left ventricle with normal cavity size and wall thickness. Systolic function was preserved with an ejection fraction of 55%. There were no regional wall motion abnormalities. The right ventricular cavity size was at the upper limits of normal, with normal systolic function. A large mass measuring greater than 5 cm was noted at the interventricular septum that projected into the left ventricular outflow tract (LVOT) (Figure 3).

**Figure 4:**
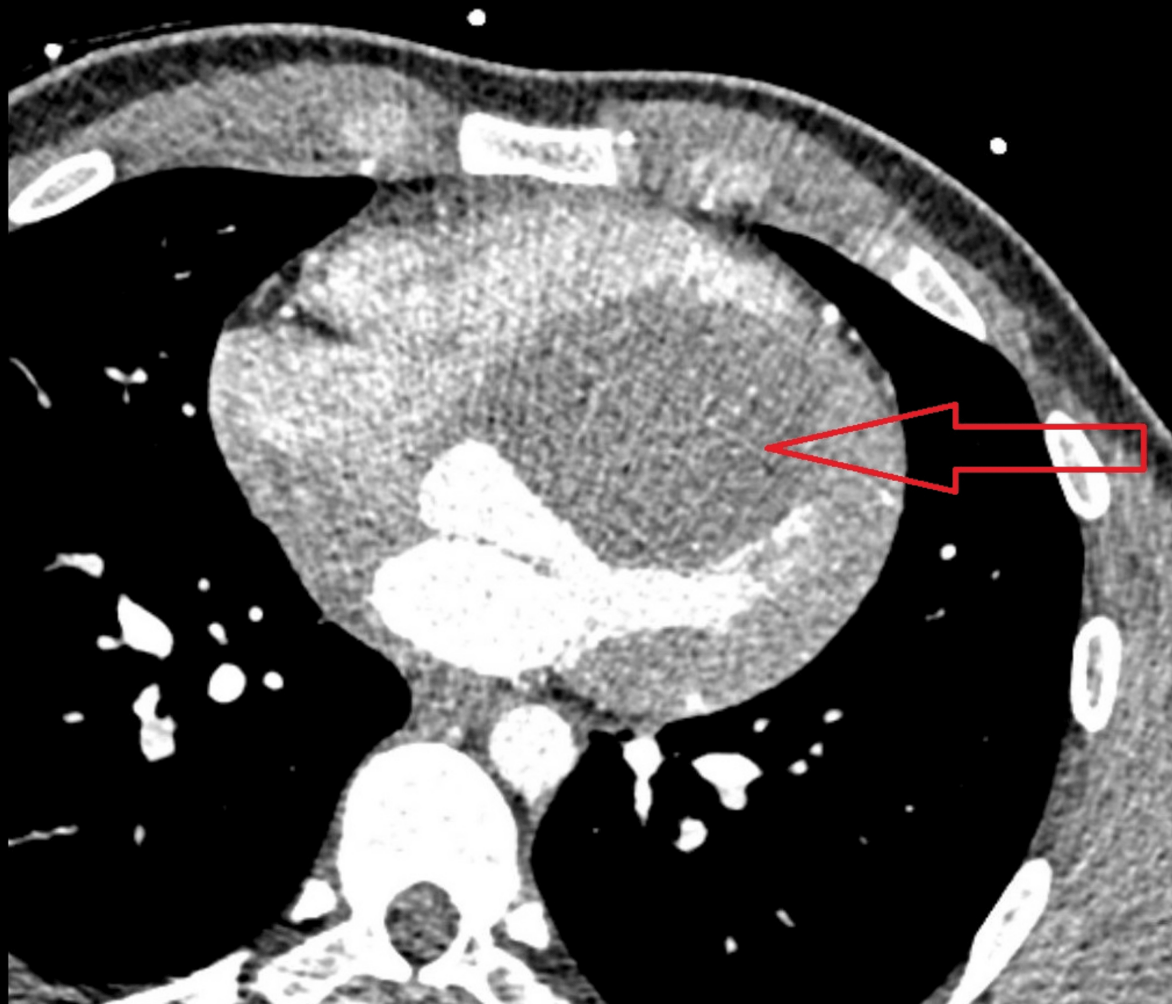
Coronary computed tomography angiography (axial view) showing a large fibroma causing LVOT obstruction LVOT, left ventricular outflow tract

A CT coronary angiogram was performed to rule out a possible anomalous coronary artery. Imaging revealed a solid mass involving the heart, which was seen predominantly along the mid to basilar segments of the anterior walls of the left ventricle and right ventricle. There was also involvement in the myocardium and pericardium with extension into the pericardial recesses. This mass was noted to be causing moderate narrowing of the aortic outflow tract. It measured 7.2 x 6.3 x 5.8 cm. In addition, there was a calcified mass in the left ventricular apex measuring 3.7 x 2.5 cm, which was suspected to be a calcified aneurysm (Figure 4). Cardiac MRI was not performed in this patient due to this service being unavailable at our hospital.

During admission, the patient was maintained on metoprolol succinate 25 mg daily for four days and had no other episodes of ventricular tachycardia on telemetry. Because the patient and his family had already declined surgical intervention, AICD placement was offered; however, he also refused this intervention after extensive discussion about the risks and benefits. He was subsequently discharged on metoprolol succinate and with continued outpatient follow-up with his cardiologist.

## Discussion

Gorlin syndrome, also known as Gorlin-Goltz syndrome or nevoid basal cell carcinoma, is a genetic condition inherited in an autosomal dominant fashion. The syndrome increases the risk of basal cell carcinomas as well as neoplasms of the neurologic, ophthalmologic, and skeletal systems. The prevalence of the disease is estimated at 1 per 40,000 to 60,000, and it is equally distributed between men and women [3].

It is caused by a mutation in the patched 1 (*PTCH1*) gene on the short arm of chromosome 9. This codes for transmembrane receptors that interact with signaling proteins of the sonic hedgehog family, which limits tumorigenesis. For patients to develop these characteristic tumors, they would need mutations in both copies of the *PTCH1* tumor suppressor gene. However, patients generally inherit one mutated copy and develop a second hit mutation in the second copy and thus express features of the disease. Gorlin syndrome can also be caused by mutations in the patched 2 (*PTCH2*) gene and suppressor of fused (*SUFU*) gene as well [3].

Other clinical manifestations of the syndrome include seizures, developmental abnormalities, splayed ribs, frontal bossing, cleft lip/palate, pectus excavatum, and syndactyly. Patients can also have pathological manifestations such as colobomas, strabismus, cataracts, and hypertelorism [3]. Our patient is noted to have a congenital cardiac fibroma, which is present in 3% to 5% of patients with Gorlin syndrome. Primary cardiac fibromas make up 1% of all primary cardiac tumors. They are more common in children and are rare in adults; however, they can cause significant complications. This is due to the fact that they can grow to relatively large sizes that cause significant mass effects and disrupt the heart’s conduction pathways. These manifestations include left or right ventricular outflow obstruction, coronary artery compression, valvular regurgitation, and arrhythmias [4,5].

Although cardiac fibromas are usually asymptomatic, they are particularly arrhythmogenic when compared to other primary cardiac tumors. This is hypothesized by some to be due to not only the mass effect but also because, at the histological level, there are interdigitating areas of the myocardium that act as the substrate for these arrhythmias [4,5]. Our patient represents a perfect example of this as he denied any previous history of cardiac symptoms and was never intolerant of physical activity but has now presented with sustained ventricular tachycardia.

Certain factors portend a poor prognosis. One of these is the location of the tumor. Tumors located in the interventricular septum are noted to be associated with a greater risk of sustained arrhythmias, likely due to the proximity of the Bundle of His, right and left bundles, as seen in our patient. A higher tumor-to-heart ratio is also a poor prognostic indicator, which is related to the fact that tumors that are large relative to the heart size will have greater hemodynamic effects [5].

Cardiac fibromas are not known to regress like other tumors such as rhabdomyomas, as seen with our patient who has had this tumor since birth. Due to this fact, surgical resection should be of utmost consideration in these patients [3,4]. Our patient declined to have the surgical removal of the tumor in the past due to personal preference and continued to decline during this admission as well for similar reasons. Alternatively, the patient was offered AICD placement for secondary prevention of ventricular tachycardia, as this treatment option is usually offered to patients with inoperable cardiac fibromas [4]. However, he declined to have AICD placement as well.

## Conclusions

Although ventricular tachycardias have a wide range of causes, this case reminds us that structural causes including cardiac tumors are also an important albeit rare cause. It is important that this is included in our differential especially in young patients presenting with ventricular tachycardia. These arrhythmias are caused by the compression of the cardiac conduction system by the adjacent mass, interdigitating areas of myocardium as well as accompanying fibrosis. Imaging is important in these cases because it allows us to rule out differentials and identify important complications such as LVOT obstruction. Cardiac fibromas tend to be asymptomatic, but they are particularly arrhythmogenic and pose a great risk to patients if not excised or if AICD is not placed to prevent mortality. However, some patients may remain stable with medical therapy alone.
